# 

**Published:** 1905-12-15

**Authors:** 


### ANNOUNCEMENTS.

DEATHS OF OHIO DENTISTS. Dr. E. L. Bone of Dayton, died October 22d, 1905; Dr. M. L. Williamson, of Marietta, was drowned October 22d, 1905; Dr. E. C. Lentz, of Columbus, died October 8th, 1905.

DEATHS OF MICHIGAN DENTISTS. Dr. Henry F. Boultes, of South Haven, died in Pensacola, Florida, October 3d, 1905, of yellow fever; Dr. O. D. Jones, of Marquette, died October 19th, 1905; Dr. Victor E. Tuttle, of Ann Arbor, died September 5th, 1905, of apoplexy.

MARRIED.—Dr. Thomas T., Moore, jr., of Columbia, S. C., was married November 8th, 1905, to Miss Kate L. Crawford, at Columbia. The REGISTER and its readers extend hearty congratulations to young Tom for the sake of old Tom.

VISITING LIST.—We have received a copy of the annual Physician's Visiting List, published by P. Blakiston's Son & Co. It is as usual, well made, convenient, and contains much information in convenient and compact form for ready reference. Several additions are made to accommodate small or large practices.

Dr. H. M. Bridgeman, of Kimberly, S. A. has just returned to South Africa after spending the summer in this country with his family. The doctor is an enthusiastic professional man and spent much of his time in acquainting himself with the progress made in dental art during the last seven years while he has been in Africa. He thinks his adopted country has a great future, but regrets that American professional men are not more cordially welcomed by the English laws regulating practice.

American Graduate Locates in Germany. Dr. Eugene F. Strom, graduate of the Chicago Dental College in 1904, and the University of Michigan, 1905, has located in his native town of Landau, Germany. The local practitioners have not welcomed him professionally, but are endeavoring to discredit his preparation. He is a thoroughly prepared man and will undoubtedly win out.

South Dakota Examiners. This Board will hold its next meeting at Sioux Falls, January 16th, 1906. Applicants should correspond with the secretary, Dr. G. W. Collins, Vermillion, S. D., before January 9th, for full particulars.

Indiana Board of Examiners. This Board will hold its next meeting at Ft. Wayne, Ind., January 9th to 11th, in the office of Dr. J. S. McCurdy. Applications must be filed with Dr. F. R. Henshaw, Middletown, Ind., not later than January 4th, 1906.

INSTITUTE OF DENTAL PEDAGOGICS. The annual meeting of this society will be held in the Fifth Avenue Hotel, New York City, December 28th to 30th. While this society is organized for the discussion of teaching methods, its proceedings are of great interest and value to the practitioner. The meeting this year will undoubtedly be most interesting because of the subjects to be discussed and the men who are to contribute.

A NEW EXAMINER FOR MICHIGAN. The Governor has appointed Dr. B. L. LeGro of Three Rivers, to succeed Dr. C. J. Gray as a member of the State Board of Dental Examiners for Michigan. Dr. Gray has been a very efficient member of the Board and has done good work on the Board. To his work is due the reciprocity relations with New Jersey and the Northwest, Canadian Territories. Dr. LeGro is a graduate of the University of Michigan and has a good practice at Three Rivers, Mich. He is a good professional man, a scholar and full of energy and will doubtless do effective work in supporting the legal standards for practice in the State.

